# Experimental Investigation on Diamond Band Saw Processing of Resin Mineral Composites

**DOI:** 10.3390/ma17081814

**Published:** 2024-04-15

**Authors:** Jiahao Sun, Jianhua Zhang, Weizhou Gu, Yunfang Long, Chuanxin Guo

**Affiliations:** 1 Key Laboratory of High Efficiency and Clean Mechanical Manufacture, School of Mechanical Engineering, Shandong University, Jinan 250061, China; 202320655@mail.sdu.edu.cn (J.S.); 202314327@mail.sdu.edu.cn (W.G.);; 2 National Demonstration Center for Experimental Mechanical Engineering Education, School of Mechanical Engineering, Shandong University, Jinan 250061, China; 3 Jinan North Jinfeng Saw Industry Co., Ltd., Jinan 250061, China; jinfengjuye@163.com

**Keywords:** resin mineral composites, diamond band saw, sawing force, machined surface morphology, processing defects

## Abstract

Resin mineral composite (RMC) is a new material with several times the damping properties of gray cast iron and great corrosion resistance. Due to its overall brittleness, sawing with a diamond band saw would be a suitable method. In this research, sawing experiments are carried out to study the sawing force characteristics of the material and its surface morphology during the processing. The results show that the feed force level is in the range of 3.5~5.5 N and the tangential force level is relatively low. The distribution of resin mineral components does not have a significant impact on the average sawing force but increases the fluctuation of the lateral force signal. The maximum fluctuation volume is 94.86% higher than other areas. Uneven lateral force, generated when diamond particles pass through the resin–mineral interface, is one of the causes of fluctuations. The machined surface of RMC has uniform strip scratches and a small number of pits. Maintaining a constant ratio of sawing speed to feed speed can result in approximately the same machined surface. A step structure with a height of about 10 μm appears at the interface of resin minerals. As a processing defect, it may affect the performance of RMC components in some aspects, which need a further precision machining processing.

## 1. Introduction

As the main work machine in the manufacturing industry, CNC machine tools have an irreplaceable position in industrial manufacturing. With the complexity of equipment in aerospace, automotive, medical, and other fields, the industry has put forward increasingly higher requirements for the performance of CNC machine tools [1,2]. The use of new materials with a superior vibration resistance and high strength is an important way for machine tool manufacturing to become high-end [3]. Resin mineral composite is prepared with minerals such as granite as an aggregate and organic resin as a binder. It has the ability to form and integrate complex shapes [4]. At the same time, the damping properties and corrosion resistance are also excellent. It can have high stiffness while maintaining low density. Based on the above advantages, it has been widely used in components of precision machine tools, electronics, medical, and other equipment. These applications can improve the stability of equipment under high-speed operation [5,6].

Exploring the material properties of RMC and improving its performance through optimizing the preparation process have always been research hotspots in the industry. C Zhang et al. [7] improved the mechanical properties of RMC by adding Mo fiber. The effects of the fiber surface state and hardener content on the RMC interface bonding strength and mechanical properties were studied. Aneli J et al. [8] modified basalt minerals with tetraoxysiloxane and prepared polymer composites. The effects of the modification process on the ultimate strength, softening temperature, and water absorption of the composites were studied.

RMC is a typical material composed of multiple components. Due to the different physical properties of each component, it is more prone to defects during processing [9]. Scholars reported this phenomenon when studying the processing of similar composite materials. Qi et al. [10] discovered significant differences in the pore diameters of different materials when studying pore-making in CFRP/Ti laminated materials. They called it the stepped hole defect. Sasahara H et al. [11] found that different fiber orientations can significantly affect the surface quality and tool wear when processing CFRP with a circular saw. If the fiber orientation was −45°, the machined surface would appear to be very rough. In addition to the complexity of the ingredients, RMC also has a high brittleness, which has caused it to inevitably accompany many processing problems [12]. Considering that RMC is often used in components with smooth surfaces, high linear requirements, and high size accuracies, it is necessary to develop efficient and precise processing methods that can obtain high-quality surfaces. It will promote the further engineering application of RMC [13]. At present, when facing RMC components with higher quality requirements, the method often adopted is to rough-cut first and then polish with a grinding wheel. This method is inefficient. And, if the machining allowance is not well controlled, it can easily lead to component dimensions that are out of tolerance [14]. To address this problem, we try to introduce diamond band saw technology into RMC component processing. Diamond band saw processing is an emerging processing method. It removes material by grinding through diamonds embedded in a steel strip [15,16]. Due to its advantages of energy saving and high efficiency, it is excellent for the processing of high hardness non-metallic brittle materials [17]. Through this research, we intend to verify the feasibility of precision machining with a diamond band saw and explore the process method suitable for high-quality machining of RMC.

In this research, a platform is built to carry out experiments on processing RMC with a diamond band saw. The sawing force characteristics and surface quality during the sawing process are tested. Through comprehensive experimental analysis and finite element simulation methods, the effect of process parameters on sawing force is explored. The correlation between force signal fluctuations and resin–mineral composition distribution is revealed. The surface morphology characteristics of RMC processing are clarified. Meanwhile, the existence of step-shaped processing defects at the resin–mineral interface is discovered. Through the research, it aims to provide a new idea for the precision processing of RMC and promote the wider application of RMC components in advanced instruments.

## 2. Materials and Methods

### 2.1. Materials

**Figure 1:**
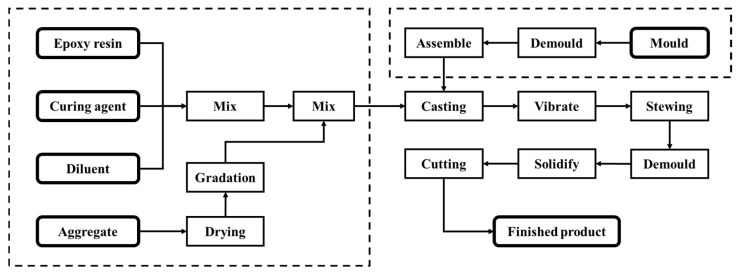
Preparation process of resin mineral composites.

RMC workpieces were produced through the process flow shown in Figure 1, which is divided into three main steps: raw material mixing, mold preparation, and casting.

The base material of RMC was bisphenol A epoxy resin and its grade was 618A. During preparation, curing agent, diluent and fly ash needed to be added to the resin. The curing agent used in the experiment was 593 curing agent, which was the adduct of diethylenetriamine and butyl glycidyl ether with a molecular weight of 217.13. In order to ensure that the cured product had sufficient strength, the mass fraction of the curing agent was set to 20%. The epoxy value of the material was controlled to around 0.51 by adding diluent. The aggregate components of RMC were Jinan blue granite and quartz stone. Their mechanical properties are shown in Table 1. For aggregates, the surface of the aggregates was treated with a silane coupling agent in advance to improve the interface bonding state between the aggregates and the epoxy resin. The volume ratio of the coupling agent, deionized water, and absolute ethanol was 1:1:8. The coupling treatment temperature was 20 degrees Celsius and the treatment time was 10 min. After processing, the resin was mixed with a graded aggregate and stirred thoroughly.

After mixing the various component materials, they were cast with the help of a prepared mold. Considering that the existence of voids will affect the mechanical properties of RMC, a composite defoaming process was introduced to reduce the porosity. The specific method was to first use 10 °C defoaming agent in conjunction with the pressure plate casting process. Among them, the defoaming agent was dimethyl silicone oil with a mass fraction of 1.5%, and the mass of the pressing plate was 1 kg. Then, after constant temperature curing, demolding, room temperature curing, and other steps, the finished RMC workpieces were obtained. The size of the completed RMC workpieces was 160 × 40 × 40 mm^3^. After testing, the mechanical properties and other indicators of RMC met the usage requirements.

### 2.2. Experimental Method

**Figure 2:**
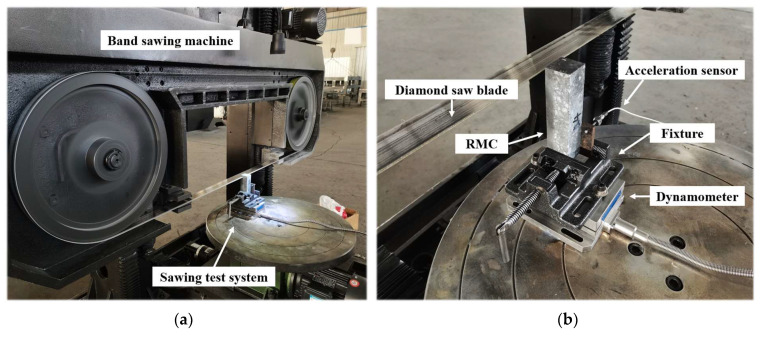
Diamond band saw-cutting experiment: (**a**) diamond band sawing machine; (**b**) sawing test system.

The sawing experiment was carried out on the W-900 band saw machine produced by Jinan North Jinfeng Saw Industry Co., Ltd., (Jinan, China) as shown in Figure 2a. The sawing machine was equipped with a diamond band saw blade, the specification of which is 4800 × 50 × 0.65 mm^3^. The diamond band saw blade is a composite steel strip made by electroplating diamond particles on spring steel. It can cut hard materials by grinding the material with diamond particles. The tooth shape of the diamond band saw blade used in the experiment was continuous teeth. This tooth shape has a wide range of uses and has the advantages of a smooth cutting surface as well as low material loss. The mesh number of the band saw blade was 80.

The RMC workpieces were fixed on the dynamometer through a special fixture to form the sawing test system, as shown in Figure 2b. The model of the dynamometer was Kistler 9257B (Kistler Group, Winterthur, Switzerland). It consisted of four internal force sensors that could measure three orthogonal components of force. The eight-channel mode of the dynamometer was used during the experiments. Based on the internal measurement principle, the three-directional force calculation method can be obtained: superimpose the output signals of channel 1 and channel 2, which is the force *F*_x_ in the x direction; superimpose the signals of channel 3 and channel 4, which is the force *F*_y_ in the y direction; and superimpose the signals from channels 5 to 8 is the force *F*_z_ in the z direction. Through the above method, the tangential force (*F_t_*), feed force (*F_f_*), and lateral force (*F_l_*) during the processing can be obtained. The sawing test system was also equipped with a Kistler 8763B050BB three-axis acceleration sensor (Kistler Group, Winterthur, Switzerland), which was connected to the clamp using bolts. It was mainly used to collect the vibration of the fixture during processing to ensure that the workpieces were firmly clamped and the workbench did not shift.

**Figure 3:**
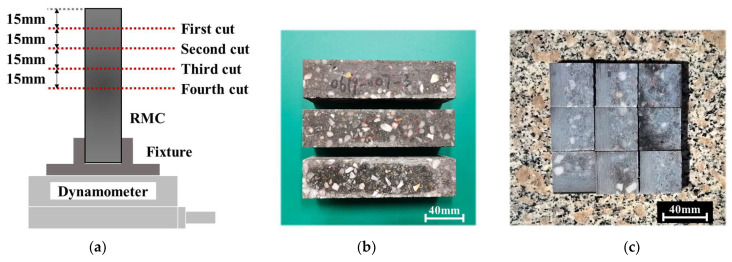
RMC workpieces: (**a**) sawing position; (**b**) prepared workpiece; (**c**) slices obtained by sawing.

During the experiment, each workpiece was cut four times under the same process parameters. The cutting positions are shown in Figure 3a. For the same workpiece, the workbench needed to be reset every time sawing was completed. Meanwhile, the saw frame was lowered to a certain height through the CNC system before performing the next sawing operation. In this way, the thickness of each slice was guaranteed to be 15 mm. The processed slices are shown in Figure 3c. After the processing of a single workpiece was completed, the experimental parameters were modified and a new workpiece was replaced. Then, the above process was repeated. A total of twelve sets of experimental parameter combinations were set up in the study, including four sawing speed level values and three feed speed level values, as shown in Table 2.

**Figure 4:**
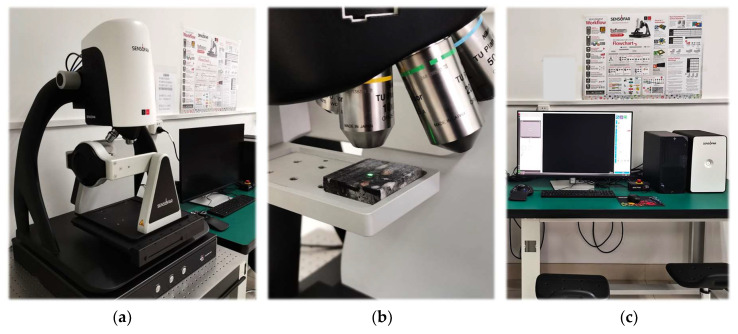
3D Optical Profiler: (**a**) device host; (**b**) microscope lens; (**c**) control panel.

After the sawing experiment, the obtained slices were placed on the Sensofar S neox 3D Optical Profiler (Sensofar Group, Terrassa, Spain). The surface morphology of the slices was observed using the laser confocal test mode, as shown in Figure 4. The lens used is 10×. Its numerical aperture (NA) is 0.3 and working distance (WD) was 17.5 mm. In each test, data was collected nine times in the 3 × 3 range and the surface morphology results were obtained by splicing the obtained data with the overlap of 30%.

## 3. Results

### 3.1. Sawing Force Characteristics

#### 3.1.1. Characteristics of Tangential Force and Feed Force

**Figure 5:**
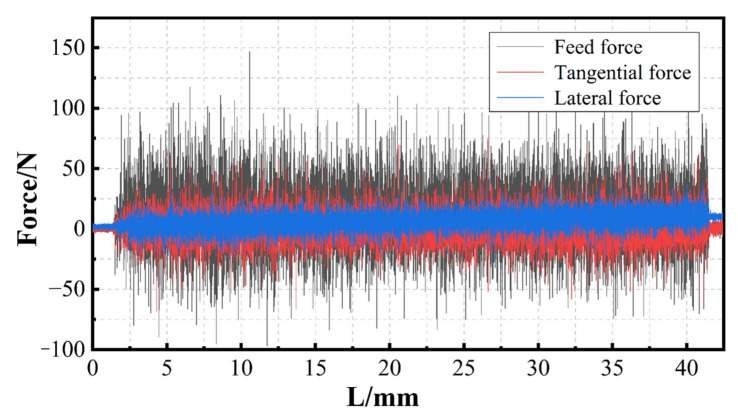
Raw sawing force signals.

The typical raw sawing force signal collected by the dynamometer is shown in Figure 5. It consists of three parts: tangential force, feed force, and lateral force. 

**Figure 6:**
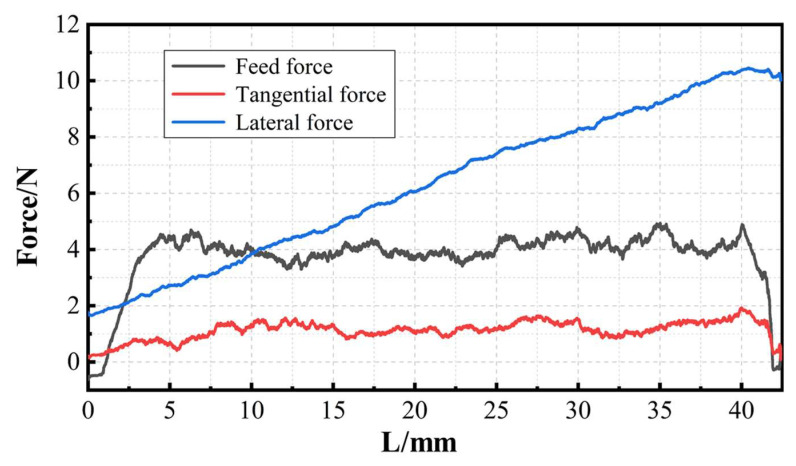
Processed sawing force signals.

The original force signal contains a certain noise and needs to be denoised. The fast Fourier transform filter is first used to perform low-pass filtering on the force signal. The cutoff frequency is set to 5 Hz. Then, the force signal is smoothed by the neighbor averaging method and the number of window points is set to 500 [18]. The processed force signals in three directions are shown in Figure 6.

It can be seen from Figure 6 that the tangential force and feed force signal have three obvious change processes. This corresponds to the three stages of the sawing process: the cutting-in stage, stable sawing stage, and cutting-out stage. In the cutting-in stage, the band saw gradually comes into contact with the workpiece and the force signal rises rapidly from 0. When the zone covered with diamonds completely cuts into the workpiece, the force value gradually stabilizes and floats in a small range. At this point, the processing enters the stable sawing stage. In this stage, the distribution ratio of resin and minerals in different depths is different. And, because their physical properties are different, the resistance of the materials when being cut off will be unequal. This becomes one of the main reasons for the floating value. However, since the components of RMC are mixed relatively evenly, the effect of mineral distribution on the average force is relatively limited. The cutting-out stage is at the end of the processing process, the slice separates from the workpiece and the force value decreases rapidly. Throughout the cutting process, the stable sawing stage occupies the longest time and it is also the stage most closely related to sawing quality and efficiency. The time of the cutting-in stage depends on the speed of feed and the cutting-out stage stops with the natural separation of the slice. Therefore, in the case of a small feed speed, the duration of the cutting-in stage is slightly longer than the time of the cutting-out stage.

Since the stable sawing stage is highly representative, the average value of the force curve in this stage is taken as the force value corresponding to the process parameter. The results of the sawing experiments under various process parameter combinations are shown in Table 3.

**Figure 7:**
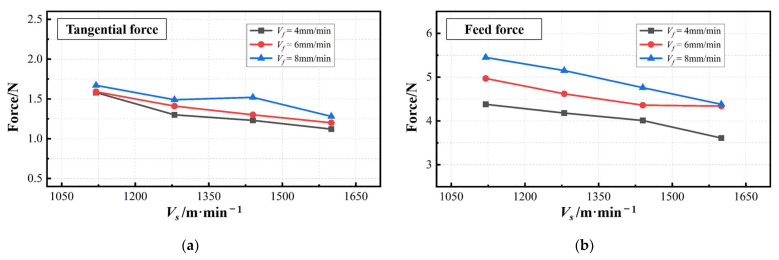
The changing trend of sawing force with process parameters: (**a**) tangential force; (**b**) feed force.

Figure 7 shows the trend of tangential force and feed force with the process parameters. As can be seen from the figure, most of the feed force is in the range of 3.5~5.5 N and its overall level is higher than the tangential force (ranging from 1.0~2.0 N). The tangential force and feed force are negatively correlated with the sawing speed and positively correlated with the feed speed. Taking Figure 7b as an example, when the maximum feed speed (8 mm/min) is matched with the minimum sawing speed (1120 m/min), the average feed force is the largest, 5.45 N. The smallest average (3.61 N) appears in the experimental group with a sawing speed of 1600 m/min and a feed speed of 4 m/min. Generally speaking, when the feed speed is certain, the increase in the sawing speed represents the increase in the number of diamond particles participating in the sawing cut. It reduces the thickness of the maximum undeformed chip of single diamond. The cutting power consumption of the unit time is reduced. Therefore, the value of the force is reduced. When the sawing speed is certain, the increase in the feed speed will increase the thickness of the material being removed per unit time. This leads to an increase in friction between the saw and the workpiece and introduces greater saw-cutting resistance, which in turn, leads to the improvement of force value.

#### 3.1.2. Characteristics of Lateral Force

Lateral force refers to the component of the sawing force that is normal to the saw surface. It is caused by the lateral vibration of the saw, as well as the collision between the saw and the surface of the RMC.

**Figure 8:**
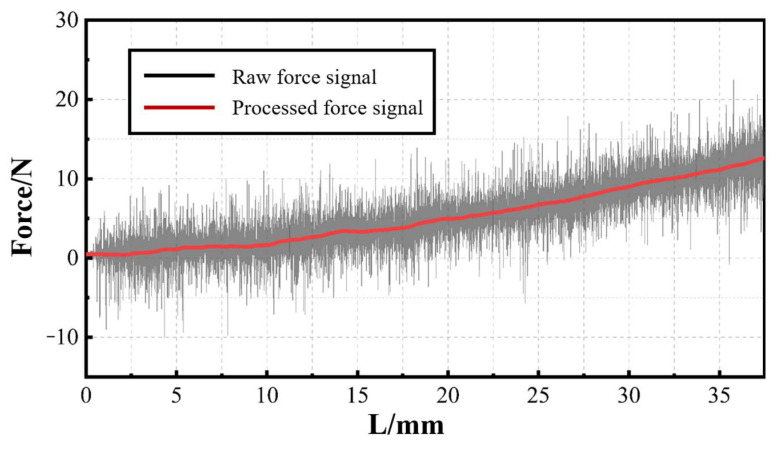
The typical raw lateral force signal and processed lateral force signal.

Figure 8 shows the typical raw lateral force signal and the processed lateral force signal. The processing method is the same as described in Figure 6. It can be seen from the figure that the lateral force shows a monotonically increasing trend as the sawing time increases. It can be understood as follows: as the sawing process progresses, the contact area between the side of the saw and the RMC continues to increase. The collision effect is positively related to the contact area. In addition, the increase in sawing depth can also easily cause poor chip removal and cause greater vibrations in the sawing part.

**Figure 9:**
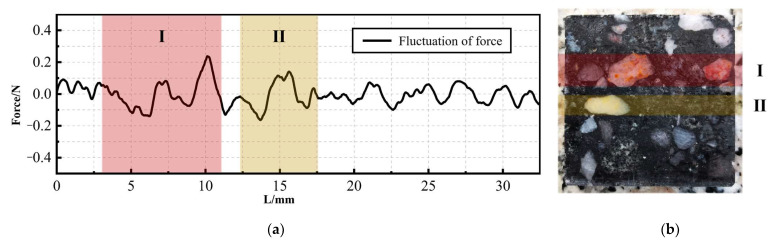
Fluctuation signal of lateral force: (**a**) fluctuation signal curve; (**b**) slice corresponding to lateral force signal.

It can also be noticed that there is a certain fluctuation in the lateral force signal. In order to explore its regularity, the existing signal is fitted to obtain a sufficiently smooth curve. The extracted difference between the two signals is the fluctuation signal, as shown in Figure 9. It can be observed from the figure that the areas with larger fluctuations on the curve are Ⅰ and Ⅱ. They correspond to the areas on the section where the mineral content and volume are larger. The absolute value of the force signal is taken as the fluctuation amount. Calculate the average fluctuations in Ⅰ, Ⅱ, and other areas, respectively. Through comparison, it is found that the fluctuation amount of the force signal in area Ⅰ is 90.40% higher than that of the other areas. The force signal in area Ⅱ is 94.86% higher. The above phenomenon shows that the saw will vibrate more when passing through a section with complex material composition. In addition to the material collapse caused by unstable cracks at the interface, unstable lateral forces are also an important reason.

**Figure 10:**
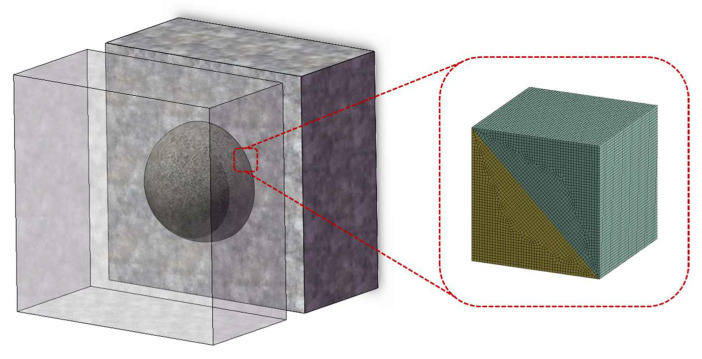
Tiny material unit at the resin–mineral interface.

In order to further explain this phenomenon, the micro-element method is used to explore the force characteristics of the diamond particle when removing materials at the resin–mineral interface [19,20]. As shown in Figure 10, the extracted tiny unit at the interface can be regarded as a cube, containing both mineral and resin materials. Meanwhile, the diamond particle is often simplified as a symmetrical bicone along a midplane. Since 50% of the particle size is buried in the saw, the outer particle can be viewed as a cone [21,22]. It passes through the material unit at different angles to achieve material removal. In fact, in a real processing environment, diamond particles also travel through the material at completely random angles.

**Figure 11:**
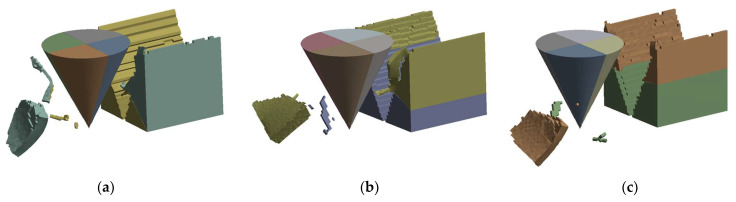
Finite element simulation results of material removal by diamond particles: (**a**) 45° angle; (**b**) 25° angle; (**c**) 0° angle.

We simulated this process using the LS-DYNA program in ANSYS. By establishing the finite element model, diamond particles traveling through three different types of material units are simulated. Each material unit is composed of two materials: resin and mineral (granite). The proportions of various materials are the same. The difference lies in the angle formed by the interface between the two materials. As shown in Figure 11, a total of 3 angles have been established, namely 0, 25°, and 45° (referring to the angle between the interface and the horizontal plane). Since diamond deformation during processing is extremely small compared to the RMC, it is set up to approximate a rigid body. The diameter and height of the conical diamond particles are 10 mm and the side length of the cube material unit is also 10 mm. In the simulation settings, the material unit is fixed. Diamond particles pass through the material unit at a constant speed with a total shift of 25 mm. Hexahedral elements are used to mesh the RMC as well as tetrahedral elements are used for diamond particles. Contact definition is body interaction and the type is eroding contact.

**Figure 12:**
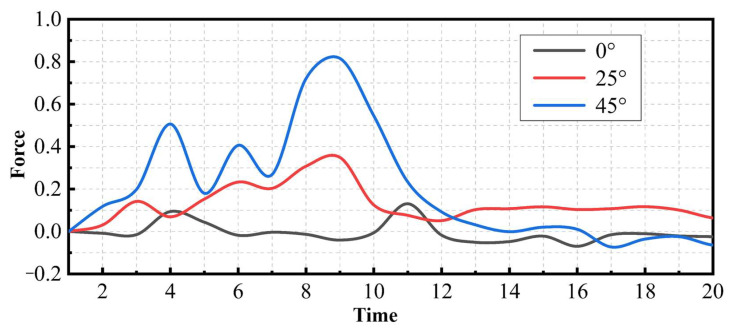
The lateral reaction force acting on the diamond particles.

Figure 11 shows the results of the simulation. The diamond particles have left V-shaped grooves in the material units. The materials that are brought out become lumps or fine chips. The preserved part of multiple units will form the morphology of the machined surface. The results obtained by extracting the lateral reaction force (force perpendicular to the direction of motion) acting on the diamond particles are shown in Figure 12. It can be seen from the figure that the reaction force also shows the characteristics of rising, stabilizing, and declining along with the three stages of processing. When the angle is 0, there is almost no lateral reaction force. As the interface angle increases, the magnitude of the reaction force increases significantly. The maximum force value of the 45° group is 133.57% higher than that of the 25° group. It is conceivable that, when a large number of diamond particles pass through the resin–mineral interface at various angles, reaction forces with different directions and values will be formed on the particles. It is reflected in the uneven and random force on the saw at the macro level. Due to the mutual cancellation of forces, the amplitude of the force will not be particularly large, but will cause fluctuations in the sawing force.

### 3.2. Processed Surface Topography

#### 3.2.1. Surface Topography of a Single Material Type Area

**Figure 13:**
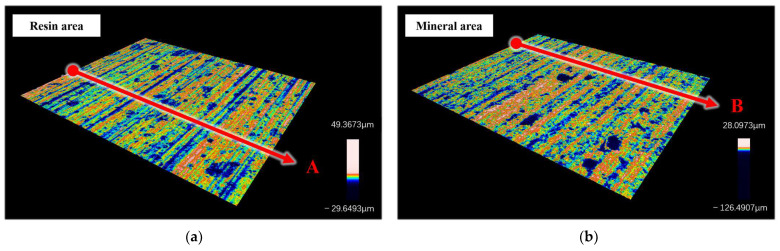
3D surface topography and section selection location (A & B) of a single material type area: (**a**) resin area; (**b**) mineral area.

Figure 13a,b show the processed surface morphology of the resin and mineral regions, respectively. It can be observed from the figure that the surfaces of both types of materials are uniformly distributed with strip scratches mixed with a small number of irregular pits. The direction of the scratches is the same as the saw’s cutting direction and perpendicular to the feed direction. Pits are often considered surface defects. Compared to resin surfaces, the area and depth of pits on mineral surfaces are generally larger. This also represents a relatively high defect rate on the mineral surface.

Strip scratches are the main feature of surface morphology. This results in material surface undulations mainly reflected in the feed direction. The characteristics of the cross-sectional features are highly random in the sawing direction. In this direction, the height difference at each position of the section is small and the height level is greatly affected by the sawing depth. Therefore, sections are taken along the feed direction to evaluate the surface quality in this research, similar to sections A and B in Figure 13.

**Figure 14:**
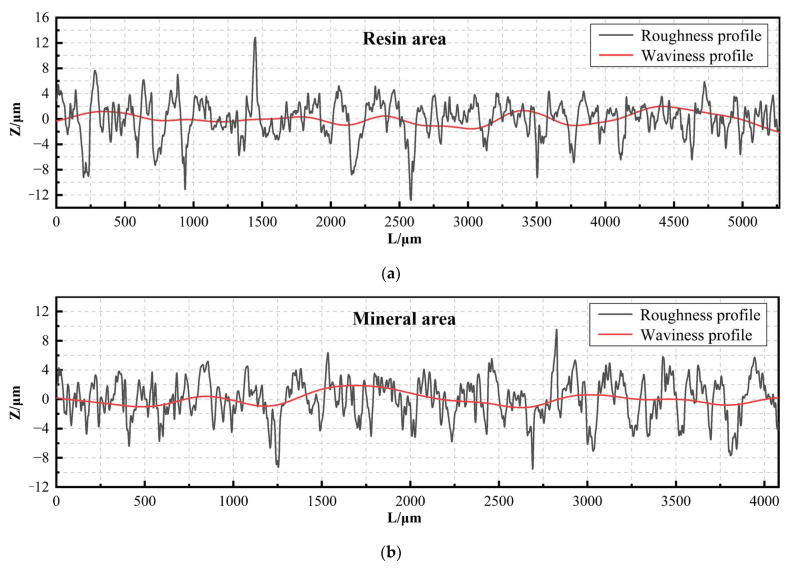
Surface roughness profile and surface waviness profile: (**a**) resin area; (**b**) mineral area.

Assuming that there is no shape error, the cross-section profile consists of surface roughness and waviness profile. Among them, waviness plays a dominant role in the periodic changes of the surface. Meanwhile, roughness is an important indicator for evaluating the fineness of surface processing. This research uses roughness as an index to evaluate the quality of sawing processing. Exporting the roughness and waviness profiles of sections A and B obtains the profile curve shown in Figure 14. According to the ISO 21920 standard [23], the corresponding roughness of sections A and B is calculated as Ra2.325 and Ra2.217.

Using the above method, four sections are taken from the surface of each sawed slice and their roughness is calculated, respectively. The obtained roughness is averaged and used as the surface roughness under the process parameters. The results corresponding to each process parameter are shown in Table 4.

**Figure 15:**
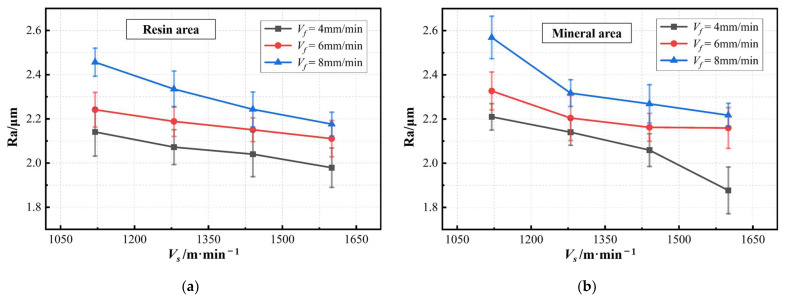
The changing trend of surface roughness with process parameters: (**a**) surface roughness of resin area; (**b**) surface roughness of mineral area.

Figure 15 is the trend of surface roughness with the changes in process parameters. Overall, the roughness of the mineral surface is slightly larger. The local collapse caused by the expansion of original microcracks in minerals will be one of the important reasons.

For both materials, the reduction of the sawing speed and the increase in the feed speed make the processing surface quality worse. This phenomenon can be explained by various reasons. On the one hand, a sufficiently high saw speed and low level of feed speed can make more diamond particles through the surface of the workpiece. This can remove the peak and is conducive to reducing roughness. On the other hand, when processing crispy materials, there is a critical cutting depth. At this depth, the material removal mechanism converts between the tough and brittle modes [24,25]. The processing surface in the tough mode is usually smoother. On the contrary, although the processing of brittle models can improve efficiency, it is easier to cause damage such as cracks and concave pits. For each single diamond particle, the higher saw speed and lower feed speed will reduce the depth of cutting. This develop processing in the direction of a more tough removal mode improves the quality of processing. The results of the experiment confirmed it. When the sawing speed is 1600 m/min and the feed speed is 4 mm/min, the best processing surfaces are obtained for both resin and mineral. At this time, their average roughness values are Ra1.979 and Ra1.877, respectively.

Low levels of feed speed are clearly beneficial in improving surface quality. However, considering that the feed speed is related to the time it takes to saw, there is a trade-off between efficiency and surface quality in actual production. It is worth noting that for the same material surface, the surface roughness values are approximately the same when the ratio of the sawing speed and feed speed (*V_s_*/*V_f_*) is constant. In this way, a similar surface morphology can be obtained [26]. Therefore, when facing the need to use a larger feed speed to increase production capacity, it can be considered to increase the sawing speed in the meantime. By doing so, the stability of sawing processing quality can be ensured.

#### 3.2.2. Surface Morphology of Resin–Mineral Interface

The interface between the mineral and resin is the weak link of the RMC. Due to the difference in the elastic strain of the materials, a certain shear stress will be generated at the interface when the force is applied. During compression or flexural failure, there are always a large number of cracks distributed along the minerals and resins. Furthermore, since the interface is mainly maintained by coupling agents, it is inherently fragile compared to continuous materials. Therefore, when material is removed and the sawing force is applied, processing defects such as micro-slip, micro-cracks, and end debonding are prone to occur. Once there are processing defects in the interface area, it will undoubtedly aggravate the failure process during use and cause the service performance of RMC components to be compromised.

**Figure 16:**
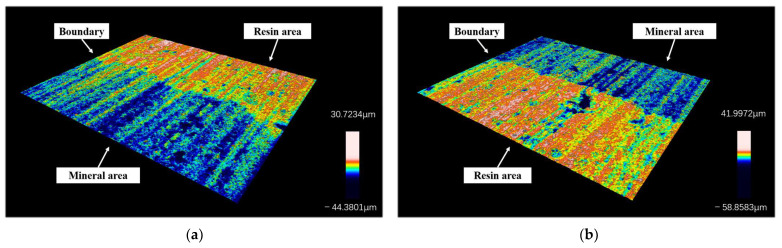
3D surface topography of resin–mineral interface: (**a**) surface under processing condition No. 1-1; (**b**) surface under processing condition No. 2-3.

In addition to the several types of processing damage just mentioned, it is also noticed in the research that the resin–mineral interface after sawing exhibits a ladder-like structure. This phenomenon is seen in almost all slices. The interfaces of two typical slices are shown in Figure 16 (taken from process parameter groups 1-1 and 2-3). It can be observed from the figure that there is a clear demarcation between the resin and mineral areas at the height level. The place of demarcation happens to be the interface.

There are a series of reasons for these phenomenon. On the one hand, minerals naturally contain internal defects and microcracks. When external forces arrive, cracks will expand and a larger volume of material will be removed. On the other hand, the thermal expansion coefficients of resins and minerals differ greatly. The resin squeezes the mineral under the heat of sawing and shrinks as it cools. It will become a potential cause of thermal damage to the interface [27]. At the same time, since the elastic modulus of minerals is much higher than resins, the minerals bear greater lateral pressure when the saw vibrates laterally. It causes the mineral to experience greater friction and more wear.

**Figure 17:**
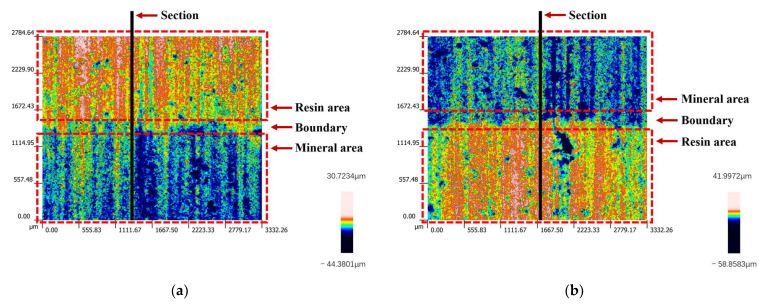
2D surface topography of resin–mineral interface: (**a**) surface under processing condition No. 1-1; (**b**) surface under processing condition No. 2-3.

**Figure 18:**
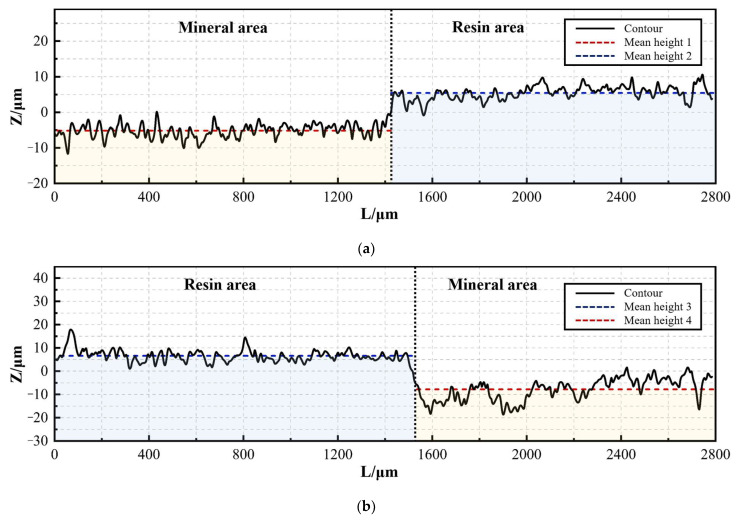
Contour curve of resin–mineral interface: (**a**) processing condition No. 1-1; (**b**) processing condition No. 2-3.

Take several sections along the direction perpendicular to the interface, as shown in Figure 17. The contour lines on the cross-section show two parts with different height levels, corresponding to the resin and mineral areas. The height levels of the two regions are averaged separately. Then, the difference between their average heights is used to obtain the height of the interface step under the contour curve. Through calculation, the heights of the ladder structures in Figure 18a,b are 10.61 μm and 14.35 μm.

A typical resin–mineral interface is found in each section, and four sections are taken from each interface. The height of the interface step is obtained using the above method and averaged, using it as the interface step height corresponding to the process parameter. The results obtained are shown in Table 5. The step height of each experimental group is maintained between 10 μm and 15 μm.

The existence of interface steps has a significant impact on the service performance of the RMC. First of all, the interface step will cause uneven load distribution during the bearing process and make it a high-incidence area for stress concentration. This will create conditions and paths for the initiation and expansion of microcracks. Secondly, an irregular surface increases friction and wear. It is easy to deteriorate the interface that already contains initial damage and cause a greater degree of shear slip. Additionally, when components are used in hot and humid environments, moisture is likely to accumulate at the interface step locations. Excessive contact time with moisture will accelerate the hygrothermal aging of the RMC, resulting in a reduction in the mechanical properties and interface bonding strength of each component material [28].

## 4. Discussion

Based on the above, the diamond band saw has achieved great results in processing RMC materials. The processing surface is relatively flat, and the surface roughness is generally below Ra2.6. The level of sawing force is maintained at a low level, showing an overall controllable trend. Meanwhile, there is no doubt that the advantages of the diamond band saw have been reflected in processing [29,30,31]. On the one hand, its shape characteristics allow it to maintain high straightness when processing large-sized components. On the other hand, since the tension has been applied on the band saw blade, it has a higher vibration resistance capacity compared to round saws and wire saws. In other words, under the same lateral force, the vibration amplitude of the band saw blade is smaller. At the same time, the feed speed related to processing efficiency is adjustable within a certain range. As long as the ratio of the sawing speed to the feed speed is constant, approximately the same machined surface can be obtained. The above advantages make it one of the most effective solutions for the processing of RMC components. Especially when the long-cut RMC components, such as the guide rail are processed, it will have superior performance. On the basis of selecting appropriate process parameters, other types of components can also obtain high efficiency and high precision when using diamond band sawing.

Nevertheless, research also reveals the existing processing defects. Machining defects mainly occur at the resin mineral interface. As mentioned earlier, defects present the step-like structure. In demanding applications such as aviation and aerospace, subsequent finishing steps may still be required [32,33]. Introducing composite processing technology in diamond band saw processing may be able to suppress the generation of defects. For example, ultrasonic vibrations can be applied to the workpiece or saw frame. It causes the diamond particles to remove material in a discontinuous manner. According to the scholars’ research experience, it is beneficial for improving surface quality [34,35]. Furthermore, it may also be helpful to select a coupling agent with better performance.

In addition to the conventional RMC involved in this research, scholars have developed some new types of RMC in recent years by adding fibers to the resin, changing the type of aggregate and so on [36,37]. Different preparation methods such as raw material pretreatment and aggregate grading will also affect the material properties to a certain extent. Studying the influence of new material characteristics on processing characteristics and summarizing the processing technology scheme that can be adjusted according to the material characteristics will be the direction of further research.

## 5. Conclusions

In this research, experiments on diamond band saw processing of resin mineral composites are carried out. The sawing force characteristics and surface morphology during the processing are explored. Through the analysis of the results obtained, the following conclusions are drawn.

(1) Both the tangential force and feed force signals during the sawing process show an increasing stable-declining trend. It corresponds to the three stages of the machining process. Their values are inversely proportional to sawing speed and directly proportional to feed speed. Under the selected process conditions, the tangential force level during stable processing is within the range of 1.0~2.0 N and the level of feed force is relatively higher. The distribution of minerals does not significantly affect the average sawing force.

(2) The magnitude of the lateral force increases monotonically as the sawing progresses. At the same time, the fluctuation of the lateral force signal is affected by the mineral distribution. Processing areas with a greater mineral content and volume are often accompanied by greater fluctuations. The average fluctuation in this area is up to 94.86% higher than other areas. The simulation results show that the uneven stress on diamond particles when passing through the resin–mineral interface will be one of the causes of fluctuations.

(3) The most typical surface morphology feature of the RMC is uniformly distributed strip scratches. Furthermore, there are a few irregular pits on the machined surface. The surface roughness of a single material type area is distributed between Ra1.8~Ra2.6. The defect rate and roughness of the mineral surface are slightly larger. When the ratio of the sawing speed to feed speed (*V_s_*/*V_f_*) is constant, the obtained surface roughness values are approximately the same.

(4) There is a step structure with a height of about 10 μm at the resin–mineral interface. The height level of the resin area is greater than that of the mineral area. This phenomenon is related to the different elastic modulus, material removal patterns, etc. between the two materials. As a kind of processing defect, it may affect the service performance of RMC components in some aspects.

## Figures and Tables

**Table 1:** Mechanical properties and density of different types of aggregates.

Aggregate Type	Compressive Strength (MPa)	Flexure Strength (MPa)	Density (g/cm^3^)
Jinan blue granite	202.43	29.04	3.071
Quartz stone	141.52	22.85	2.674

**Table 2:** Settings for experimental parameter combinations.

Processing Condition No.	1-1	1-2	1-3	2-1	2-2	2-3	3-1	3-2	3-3	4-1	4-2	4-3
Sawing speed (m/min)	1600	1600	1600	1440	1440	1440	1280	1280	1280	1120	1120	1120
Feed speed (mm/min)	4	6	8	4	6	8	4	6	8	4	6	8

**Table 3:** Tangential force and feed force under different process parameters.

Processing Condition No.	1-1	1-2	1-3	2-1	2-2	2-3	3-1	3-2	3-3	4-1	4-2	4-3
Sawing speed (m/min)	1600	1600	1600	1440	1440	1440	1280	1280	1280	1120	1120	1120
Feed speed (mm/min)	4	6	8	4	6	8	4	6	8	4	6	8
Tangential force (N)	1.12	1.20	1.28	1.23	1.30	1.52	1.30	1.41	1.49	1.58	1.59	1.67
Feed force (N)	3.61	4.34	4.38	4.01	4.36	4.76	4.18	4.62	5.15	4.38	4.97	5.45

**Table 4:** Surface roughness results for a single material type area.

Processing Condition No.	1-1	1-2	1-3	2-1	2-2	2-3	3-1	3-2	3-3	4-1	4-2	4-3
Sawing speed (m/min)	1600	1600	1600	1440	1440	1440	1280	1280	1280	1120	1120	1120
Feed speed (mm/min)	4	6	8	4	6	8	4	6	8	4	6	8
Resin area roughness (Ra)	1.979	2.110	2.176	2.040	2.150	2.243	2.072	2.188	2.334	2.140	2.241	2.456
Mineral area roughness (Ra)	1.877	2.159	2.217	2.059	2.162	2.268	2.140	2.204	2.317	2.209	2.326	2.568

**Table 5:** Resin–mineral interface height difference of each experimental group.

Processing Condition No.	1-1	1-2	1-3	2-1	2-2	2-3	3-1	3-2	3-3	4-1	4-2	4-3
Height difference (μm)	11.70	13.47	12.17	11.54	12.05	14.84	10.26	12.64	13.74	13.07	12.22	13.19

## Data Availability

Data are contained within the article.
